# Programming of subthalamic nucleus deep brain stimulation for Parkinson’s disease with sweet spot-guided parameter suggestions

**DOI:** 10.3389/fnhum.2022.925283

**Published:** 2022-11-01

**Authors:** Simon Nordenström, Katrin Petermann, Ines Debove, Andreas Nowacki, Paul Krack, Claudio Pollo, T. A. Khoa Nguyen

**Affiliations:** ^1^Department of Neurosurgery, University Hospital Bern, Bern, Switzerland; ^2^Department of Neurology, University Hospital Bern, Bern, Switzerland; ^3^ARTORG Center for Biomedical Engineering Research, University Bern, Bern, Switzerland

**Keywords:** deep brain stimulation, Parkinson’s disease, programming, sweet spot, subthalamic nucleus

## Abstract

Deep Brain Stimulation (DBS) is an effective treatment for advanced Parkinson’s disease. However, identifying stimulation parameters, such as contact and current amplitudes, is time-consuming based on trial and error. Directional leads add more stimulation options and render this process more challenging with a higher workload for neurologists and more discomfort for patients. In this study, a sweet spot-guided algorithm was developed that automatically suggested stimulation parameters. These suggestions were retrospectively compared to clinical monopolar reviews. A cohort of 24 Parkinson’s disease patients underwent bilateral DBS implantation in the subthalamic nucleus at our center. First, the DBS’ leads were reconstructed with the open-source toolbox Lead-DBS. Second, a sweet spot for rigidity reduction was set as the desired stimulation target for programming. This sweet spot and estimations of the volume of tissue activated were used to suggest (i) the best lead level, (ii) the best contact, and (iii) the effect thresholds for full therapeutic effect for each contact. To assess these sweet spot-guided suggestions, the clinical monopolar reviews were considered as ground truth. In addition, the sweet spot-guided suggestions for best lead level and best contact were compared against reconstruction-guided suggestions, which considered the lead location with respect to the subthalamic nucleus. Finally, a graphical user interface was developed as an add-on to Lead-DBS and is publicly available. With the interface, suggestions for all contacts of a lead can be generated in a few seconds. The accuracy for suggesting the best out of four lead levels was 56%. These sweet spot-guided suggestions were not significantly better than reconstruction-guided suggestions (*p* = 0.3). The accuracy for suggesting the best out of eight contacts was 41%. These sweet spot-guided suggestions were significantly better than reconstruction-guided suggestions (*p* < 0.001). The sweet spot-guided suggestions of each contact’s effect threshold had a mean error of 1.2 mA. On an individual lead level, the suggestions can vary more with mean errors ranging from 0.3 to 4.8 mA. Further analysis is warranted to improve the sweet spot-guided suggestions and to account for more symptoms and stimulation-induced side effects.

## Introduction

Deep brain stimulation (DBS) is an effective treatment for advanced Parkinson’s disease (Deuschl et al., 2006; Krack et al., 2019). Leads are implanted in the basal ganglia, typically in the subthalamic nucleus, and electrical pulses are generated by an implantable pulse generator. However, finding effective stimulation parameters is currently time-consuming with monopolar review (Ten Brinke et al., 2018). Contacts are tested one at a time and current amplitude or voltage is increased in steps until the patient experiences symptom relief or stimulation-induced side effects to determine a therapeutic window for each contact (Volkmann et al., 2006).

Directional leads have been introduced in recent years that allow for steering and shaping of the stimulation field. Whereas traditional leads consist of four omnidirectional contacts, the directional leads have six or more directional contacts, greatly adding to the complexity of finding effective stimulation parameters (Schupbach et al., 2017).

As a result of the time-consuming monopolar review for directional leads, research groups have been working on identifying effective stimulation regions in the brain (“sweet spots”), whose activation has been correlated to favorable outcomes (Dembek et al., 2019; Nguyen et al., 2019).

Our goal is to improve clinical outcomes by developing an algorithm that reduces the time and complexity of DBS programming while fully exploiting the potential of directional stimulation. To this end, the objectives of this retrospective study were to:

(1). Develop algorithms to automatically suggest lead level and contact and estimate effect thresholds for each contact using a previously published sweet spot (Nguyen et al., 2019).
(2). Build a graphical user interface to run the suggestions algorithms and visualize the results.
(3). Evaluate the suggestions of the algorithms against clinical monopolar review data.

## Materials and methods

A summary of the workflow: To compute suggestions for a new lead, we reconstructed the lead’s position and orientation. We used the open-source toolbox Lead-DBS (Horn et al., 2019). Then we estimated volumes of tissue activated (VTAs) for different levels, contacts, and current amplitudes. Finally, we identified those stimulation volumes that had a *desired overlap volume* with the sweet spot to suggest the best level, contact, and effect thresholds for that lead. These sweet spot-guided suggestions were compared retrospectively with clinical monopolar reviews. We detail the estimation of VTAs and the desired overlap volume below.

### Data and patient cohort

The cohort consisted of 24 patients with bilaterally implanted DBS leads at the University Hospital of Bern. The inclusion and exclusion criteria, and surgical procedure and postoperative management were the same as previously described (Nguyen et al., 2019). The target structure for the implantation was the motor part of the subthalamic nucleus. The patients were implanted with the Boston Vercise Directional system. Pre-operative 3 Tesla magnetic resonance images and post-operative computer tomography scans were performed on all patients. Effect thresholds from monopolar reviews were available for 284 contacts, including ring-mode stimulation (i.e., activating the three directional contacts on the same level together). The monopolar review evaluated rigidity reduction and noted effect thresholds for complete rigidity reduction.

### Lead reconstruction

The leads’ position and orientation were reconstructed with the open-source Lead-DBS toolbox in Matlab 2020b (Mathworks, Natick, MA, USA). The process has been detailed previously (Horn et al., 2019). In short, the pre-operative magnetic resonance images and post-operative computer tomography scans of each patient were coregistered to each other and normalized to Montreal Neurological Institute template space. The leads were then semi-automatically reconstructed with the PaCER algorithm and a lead’s position was manually refined so that the reconstructed trajectory matched the artifact of the lead on the computer tomography scans (Husch et al., 2018). Finally, the lead orientations were estimated using the DiODe algorithm (Hellerbach et al., 2018).

### Stimulation sweet spot

**FIGURE 1:**
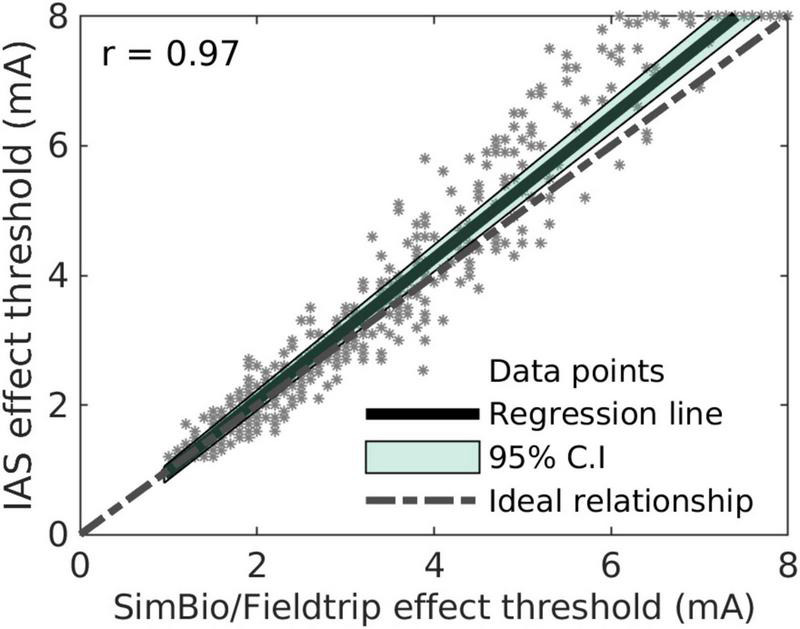
Comparison of effect threshold suggestions by the instant analytical spheres (IAS) algorithm and by the SimBio/FieldTrip algorithm.

The sweet spot for rigidity reduction used in this study is detailed in Nguyen et al. (2019). The sweet spot included voxels with efficiency scores at the 90th percentile and above and was located in the dorsolateral part of the subthalamic nucleus, Figure 1. The sweet spot was computed for the right hemisphere by pooling all stimulation volumes to that hemisphere. To have a sweet spot for the left hemisphere, the right sweet spot was non-linearly warped with Lead-DBS to the left hemisphere. Importantly, the sweet spot had been calculated with a previous and different cohort from our center than the cohort tested in this study.

### Estimation of volume of tissue activated

The volumes of tissue activated were estimated with two algorithms. The first algorithm was the SimBio/FieldTrip pipeline in Lead-DBS (Horn et al., 2019). Conductivity values for gray and white matter were set to 0.33 and 0.14 S/m, respectively. Gray and white matter were assigned with the DISTAL atlas (Ewert et al., 2018). The potential gradient was thresholded at 0.2 V/mm to get the binarized VTA (Astrom et al., 2015). The second algorithm was called *Instant Analytical Spheres* that we developed for this study to accelerate computation. The SimBio/FieldTrip algorithm can take a few minutes per VTA. The instant analytical spheres algorithm estimated VTAs markedly faster by assuming VTA and sweet spot to be spheres. Thus, the overlap volume between the two spheres was calculated with an algebraic expression. Both algorithms are described below. The FastField method to estimate VTAs would also have been a viable alternative (Baniasadi et al., 2020). However, a wrapper to compute VTAs without user interaction did not exist for FastField at the time. It would have required user input and would therefore have been more time-consuming.

The VTAs were estimated in the Montreal Neurological Institute template space to evaluate tests across patients. In a small cohort of three patients, we did not find significant differences between calculations in native space or template space (data not shown) and therefore chose to stay in template space.

#### SimBio/Fieldtrip-based algorithm

To suggest stimulation settings for a new lead, first, the three contacts closest to the center of mass of the sweet spot were shortlisted. Second, VTAs were generated for these three contacts from 1 to 8 mA in steps of 1 mA with SimBio/FieldTrip. An *initial* estimate *I(i’)* of the effect threshold was obtained by selecting the current amplitude at which the overlap volume between the VTA and the sweet spot was closest to the desired overlap volume *V*_*d*_, i.e.:


(1) $$i^{\prime }=\underset{i}{\text{argmin}}|V\left(i\right)-V_{d}|,\text{where}i=1,2,\ldots ,8\mathrm{mA}$$

where *i’* was the stimulation index of the initially estimated effect threshold, *V(i)* was the volume of VTA-sweet spot overlap at index *i*, and *I(i)* was the current at index *i*.

Third, a *refined* estimate of the effect threshold was calculated because the step size of 1 mA provided only a rough, initial estimate. A linear interpolation was performed between *[I(i’),V(i’)]* and *[I(i’+1),V(i’+1)]* for underestimated initial estimates, i.e., between the current amplitude and the overlap volume. For overestimated initial estimates, a linear interpolation was instead performed between *[I(i’),V(i’)]* and *[I(i’-1),V(i’-1)].* The refined estimate of the effect threshold *I*_*est*_ was then obtained as the current amplitude corresponding to an overlap volume of *V*_*d*_ based on the linear interpolation.

#### Instant analytical spheres algorithm

We developed a mathematical model called *instant analytical spheres* to accelerate computation, where both the sweet spot and the VTA were assumed to be spheres. This led to an algebraic expression for the overlap volume:


(2) $$V_{O}\left(r_{S\,S},d,r_{V\,T\,A}\right)=\frac{\pi }{12\,d}({\left(r_{V\,T\,A}+r_{S\,S}-d\right)}^{2}$$

$$\left(d^{2}+2d\left(r_{V\,T\,A}+r_{S\,S}\right)-3{\left(r_{V\,T\,A}-r_{S\,S}\right)}^{2}\right))$$

where *V*_*O*_, *r*_*SS*_, *d* and *r*_*VTA*_ were the overlap volume, radius of the sweet spot, distance between the sweet spot center and VTA center, and radius of the VTA, respectively. Note that Eq. (2) only held when the VTA and sweet spot intersected, i.e., |(*r*_*VTA*_−*r*_*SS*_)| ≤ *d* ≤ *r*_*VTA*_ + *r*_*SS*_, and the sweet spot was *not* completely inside the VTA.

As Eq. (2) expressed the overlap volume in terms of the VTA radius and not the current amplitude, a transformation between these two variables was required. The transformation was found empirically by generating VTAs with the SimBio/FieldTrip algorithm from 0.6 to 10 mA in steps of 0.1 mA for a single omnidirectional contact, calculating the radius corresponding to each VTA, and fitting a second-degree polynomial with intercept zero. The transformation was then validated on one omnidirectional contact and one directional contact of another patient and hence used for the remainder of the analysis.

Next, while estimating directional VTAs, we observed that the center of the VTA moved as the current amplitude increased. More specifically, the position vector **p**_*VTA*_ of the center was assumed to be (bold typeface indicating a three-dimensional vector):


(3) $${\text{p}}_{\mathrm{VTA}}={\text{p}}_{e}+\gamma \,\frac{{\text{p}}_{e}-{\text{p}}_{c}}{\left|{\text{p}}_{e}-{\text{p}}_{c}\right|}\,r_{V\,T\,A}$$

where p_*e*_ was the position vector to the center of the contact’s surface, p_*c*_ the position vector to the center of that lead level (both vectors were extracted from the lead reconstruction in Lead-DBS), and ɣ a parameter that determined how the VTA center moved with increasing current amplitude. The default value of ɣ was set to 0.1, meaning that for each millimeter increase in VTA radius, its center moved 0.1 mm further on that trajectory. For omnidirectional stimulation, on the other hand, the VTA center was assumed to be at the center of the lead level for all current amplitudes.

For the spherically approximated sweet spot, we verified that the center of the presumed spherical sweet spot was identical to the center of the original sweet spot. Together, these steps yielded the necessary radii and distance to compute the overlap volume with Eq. (2).

Finally, to suggest the effect threshold, the algorithm selected the current amplitude at which the overlap volume reached the desired overlap volume, *V*_*d*_. This was identical to the SimBio/FieldTrip-based algorithm.

We validated the instant analytical spheres algorithm in two steps. First, we evaluated the spherical approximations with the Dice similarity coefficient:


(4) $$D\,i\,c\,e\,s\,i\,m\,i\,l\,a\,r\,i\,t\,y\,c\,o\,e\,f\,f\,i\,c\,i\,e\,n\,t\,=2\,\frac{\left|V_{1}\,\cap V_{2}\right|}{\left|V_{1}\right|+\left|V_{2}\right|},$$

where *V*_1_ and *V*_2_ were two VTAs or one VTA and the sweet spot (for instance, *V*_1_ with the instant analytical spheres algorithm and *V*_2_ with the SimBio/FieldTrip algorithm). Second, we compared the suggested effect thresholds of the instant analytical spheres algorithm with the suggested effect thresholds of the SimBio/FieldTrip pipeline. The effect thresholds of 384 directional contacts and 96 omnidirectional contacts were estimated with both algorithms. Pearson correlation was performed to investigate the relationship between the two algorithms’ suggestions.

### Sweet spot-guided suggestions

#### Desired overlap volume

At the beginning of this study, we assumed the desired overlap volume *V*_*d*_ to be *constant* on the premise that a certain volume of the sweet spot needed to be activated for therapeutic effect. For the constant desired overlap volume, we estimated a desired overlap volume of 33% of the sweet spot. This estimate was derived from our previous publication (Nguyen et al., 2019), where we found a correlation between overlap and clinical improvement.

But during early prototyping, we ran into limitations with the constant desired overlap volume. Therefore, we also added a non-constant desired overlap volume, which was defined as a function of the distance between contact and sweet spot center. We analyzed the distance between contact to sweet spot and the percentage of activated sweet spot at the effect threshold for that contact (taken from the monopolar reviews). We then calculated a linear-mixed effect model with random effects on slope and intercept and the lead ID as grouping variable. This resulted in a *distance-dependent* desired overlap volume.

#### Best level and best contact suggestions

With the instant analytical spheres, the suggestions of the best level were made by estimating the effect thresholds of all four levels of lead. Directional contacts at the same level were considered together as one omnidirectional electrode, i.e., in “ring-mode.” The level with the lowest effect threshold was selected as the suggested best level.

The clinically best level for each lead was selected as the level with the lowest effect threshold from the monopolar review. In the case of more than one level having the same lowest clinical effect threshold, all these levels were selected as the best levels, as they were assumed to be equally effective in providing therapeutic effects. Sufficient data were available to obtain the clinically best level for 34 leads.

Similarly, the suggestions of the best contact were made by estimating the effect threshold for all eight contacts of a lead. These suggestions were compared with the monopolar reviews. In cases where two contacts were considered equally best by the algorithm (i.e., both had the lowest suggested effect threshold), the suggestion was classified as partially correct.

#### Effect threshold suggestions

The instant analytical spheres algorithm was used to calculate suggestions using the constant desired overlap volume and the distance-dependent desired overlap volume. These suggestions were evaluated against the clinical effect thresholds for 284 electrode contacts, including ring-mode stimulation.

#### Comparison against reconstruction-guided suggestions

The sweet spot-guided suggestions for best level and best contact were compared against reconstruction-guided suggestions. The latter suggestions are intended to emulate a clinician with access to the lead reconstructions. Such a clinician could look at the three-dimensional visualization of the lead with respect to the subthalamic nucleus and estimate a best level or contact. Here, the reconstruction-guided suggestion for the best level was the level with the shortest distance to the centroid of the subthalamic nucleus. Similarly, the reconstruction-guided suggestion for the best contact was the contact with the shortest distance to the nucleus’ centroid. The subthalamic nucleus was taken from the DISTAL atlas (Ewert et al., 2018). However, we assumed that such a visual inspection of the reconstruction would not allow for suggestions of effect thresholds.

### Statistical testing and evaluation metrics

A *p*-value less than 0.05 was considered statistically significant for rejecting null hypotheses. The confidence interval (CI) for the suggested best levels was calculated using the standard formula for proportion estimates of a population in a sample, namely:


(5) $$C\,I\,=p\,\pm \sqrt{\frac{p\cdot \left(1-p\right)}{N}}\,Z,$$

where *p*, *N*, and *Z* represent the proportion of correct predictions, the number of leads analyzed, and the Z-score (1.96), respectively. A binomial test was performed to test the sweet spot-guided suggestions against the reconstruction-guided suggestions of best level and best contact.

### Open-source graphical user interface

A graphical user interface was developed with MATLAB’s App Designer tool (Matlab 2020b). The user can run the algorithms described above and display results. The interface will be incorporated in a future version of the Lead-DBS toolbox and can be installed optionally. It can be launched from the Lead-DBS 3D viewer. The source code for the GUI and suggestion algorithms can be found at: https://gitlab.switch.ch/brain-stimulation-mapping/automatic_dbs_stim_params_selection.

## Results

### Instant analytical spheres

We first compared the instant analytical spheres algorithm developed herein with the SimBio/Fieldtrip algorithm in Lead-DBS. This allowed for faster computation of the overlap volume and thus faster suggestions. For omnidirectional VTAs, the average Dice similarity coefficient was 0.90 and indicated very good agreement between the algorithms. For directional VTAs, the average Dice similarity coefficient was 0.85. Of note, the instant analytical spheres model can—but does not need to—generate VTAs to suggest stimulation settings. It instead uses a mathematical representation of the VTAs and their overlap volume with the sweet spot.

The empirical transform from the VTA radius to the corresponding stimulation amplitude was found as follows: $I=0.43\,r_{V\,T\,A}+0.36\,r_{V\,T\,A}^{2},$ where *I* is the current amplitude in mA and *r*_*VTA*_ is the VTA radius in mm. An *R*^2^-value of 0.99 was obtained for this second-order polynomial fit of the current-radius transform to the data points of both omnidirectional and directional contacts of one test patient.

Finally, the effect threshold suggestions of the instant analytical spheres algorithm and the SimBio/FieldTrip algorithm are displayed for all contacts in Figure 1. The Pearson correlation coefficient was 0.97. The mean absolute error between the two algorithms was 0.3 mA, and a larger discrepancy was observed for larger current amplitudes. We deemed these values acceptable given the faster computation. With the SimBio/FieldTrip algorithm, the computation took about 3 h for all contacts in a bilaterally implanted patient. In contrast, it took approximately 5 s with the instant analytical spheres algorithm (AMD Ryzen9 16-core processor, 64 GB memory). We therefore used this algorithm for the suggestions below.

### Sweet spot-guided suggestions

The instant analytical spheres algorithm and sweet spot were used to generate suggestions for the best level, the best contact, and the effect thresholds of each contact. In one set of suggestions, a constant desired overlap volume was used. In a second set of suggestions, a distance-dependent desired overlap volume was used. These sweet spot-guided suggestions were compared retrospectively against clinical monopolar reviews considered as ground truth and against reconstruction-guided suggestions, which suggested the level or contact with the shortest distance to the centroid of the subthalamic nucleus.

**FIGURE 2:**
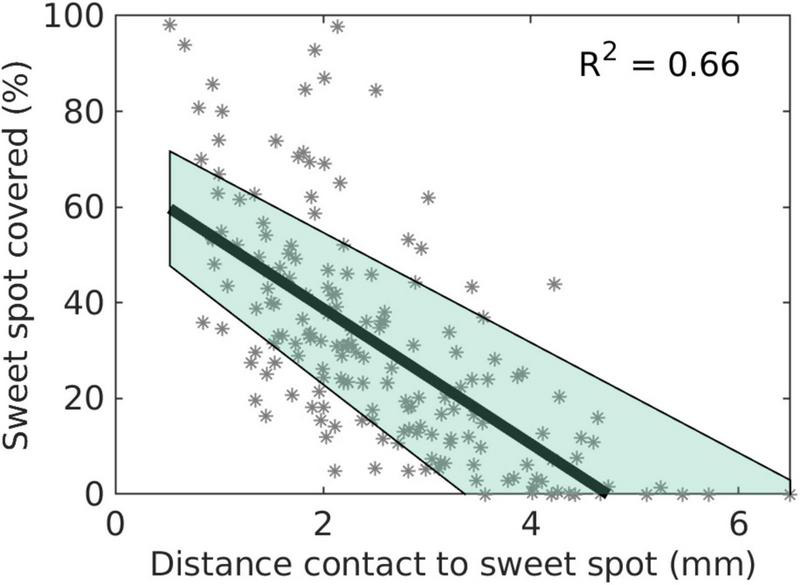
Percentage of sweet spot covered over distance between contact to sweet spot. This was to infer a distance-dependent desired overlap volume. The shaded area represents the 95% confidence interval.

To calculate the distance-dependent desired overlap volume, we analyzed the volume of the sweet spot that needed to be covered. Figure 2 shows the relationship between the percentage of the sweet spot covered and the distance contact to the sweet spot. The data was derived from the effect thresholds of the monopolar reviews. A linear mixed-effects model was fitted to the data to calculate the distance-dependent desired overlap volume. We added a lower bound of 1 mm^3^ and an upper bound of 100 mm^3^ for the distance-dependent desired overlap volume. The upper bound was chosen to be between the volume of the motor subthalamic nucleus and the volume of the full subthalamic nucleus, 62 and 164 mm^3^, respectively, according to the DISTAL atlas (Ewert et al., 2018).

For suggestions of the best level, the sweet spot-guided suggestions matched the best clinical level with an accuracy of 56% (CI: 49–73%, *n* = 34, *p* = 0.3 for a right-tailed test against reconstruction-guided suggestions of four levels, accuracy 50%). Marginally different accuracies were obtained with a constant desired overlap volume and a distance-dependent desired overlap volume.

For suggestions of the best contact, the algorithm matched the best clinical contact with an accuracy of 41% (CI: 26–56%, *n* = 39, *p* < 0.001 for a right-tailed test against reconstruction-guided suggestions of eight contacts, accuracy 13%).

**FIGURE 3:**
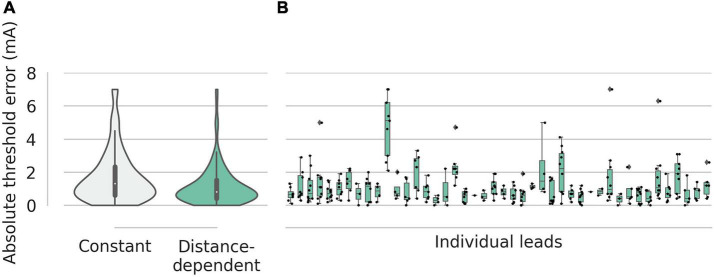
Absolute errors for the effect thresholds. **(A)** Suggestions with the constant desired overlap volume had a mean error of 1.6 mA. Suggestions with the distance-dependent desired overlap volume had a mean error 1.2 mA. **(B)** For suggestions with distance-dependent desired overlap volume, the errors of the individual leads are shown as box plots with a range of 0.3–4.8 mA.

For suggestions of the effect thresholds, we analyzed the absolute error between the clinical effect threshold and the suggested effect thresholds (Figure 3). With the constant desired overlap volume, the mean absolute error was 1.73 ± 0.09 mA. With the distance-dependent desired overlap volume, the error was smaller at 1.18 ± 0.07 mA (*p* < 0.001; two-sample *t*-test). The lead-specific errors ranged from 0.3 to 4.8 mA.

**FIGURE 4:**
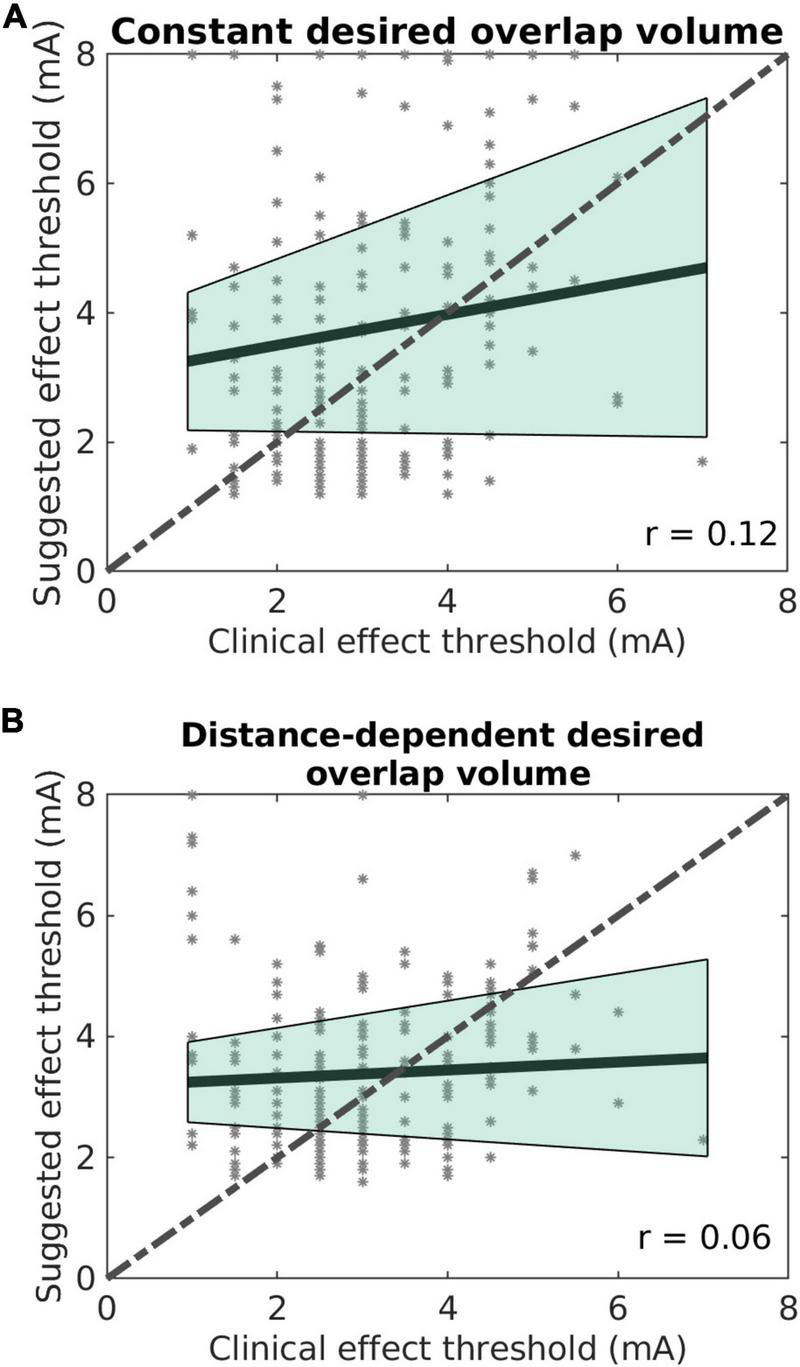
Suggested effect thresholds vs. clinical effect thresholds. **(A)** Using the constant desired overlap volume. **(B)** Using the distance-dependent desired overlap volume. The shaded areas represent the 95% confidence intervals.

But the suggestions of the effect thresholds were not well correlated with the clinical effect thresholds (Figure 4). The suggestions with the constant desired overlap volume had a Pearson’s correlation coefficient of 0.12 (*p* = 0.02) with the clinical effect thresholds. The suggestions with the distance-dependent desired overlap volume had a non-significant correlation coefficient of 0.06 (*p* = 0.2). Taken together, the sweet spot suggested the best level and contact well but not effect thresholds.

### Visualization and graphical user interface

**FIGURE 5:**
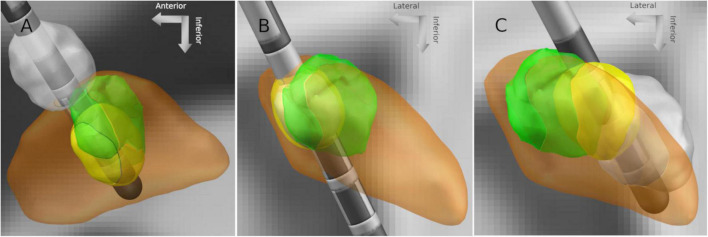
VTAs of suggested best contact and clinically best contact for three different leads **(A–C)**. Gray: clinically best stimulation setting; yellow: suggestion by the instant analytical spheres algorithm; green: sweet spot; and orange: subthalamic nucleus.

Figure 5 shows three exemplary leads with suggested contacts and effect thresholds. The suggested VTA for each lead can be seen with respect to the sweet spot, the subthalamic nucleus, the lead, and the clinically deemed best VTA for that lead. The suggestions were generated with the distance-dependent desired overlap volume.

**FIGURE 6:**
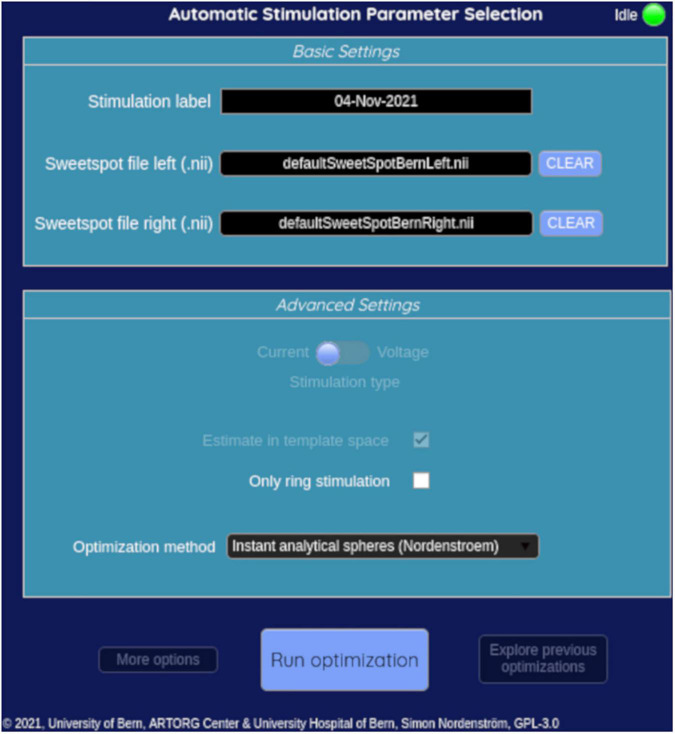
Input window of the graphical user interface.

The developed graphical user interface consists of one input window to load a user-specific sweet spot and to select the SimBio/Fieldtrip algorithm or the instant analytical spheres algorithm. This is shown in Figure 6. After computation, the results are presented in an output window. The user can select between five visualizations: (i) the suggested effect thresholds, (ii) the distance between contacts to the sweet spot center, (iii) the overlap ratios as a function of current amplitude, (iv) the overlap volume of the sweet spot, and (v) the volume of VTA *not* overlapping with the sweet spot. The selected sweet spot and the suggested VTAs can be added and displayed in the 3D viewer of Lead-DBS.

## Discussion

### Suggestions of best level, best contact and effect thresholds

In the present study, we developed an algorithm to automatically suggest the best level, the best contact, and the effect thresholds of a directional lead. The suggestions were generated in a few seconds and were guided by a sweet spot for rigidity reduction in the subthalamic nucleus. The suggestions of best level and best contact matched the best clinical levels and contacts well, whereas the suggestions of effect thresholds proved more challenging. Our algorithm is publicly available and can be added to the toolbox Lead-DBS.

The sweet spot-guided suggestions of the best level matched the best clinical levels in 56% of leads and were not significantly better than reconstruction-guided suggestions. On the other hand, the sweet spot-guided suggestions for best contact matched the best clinical contact in 41% of leads. This was significantly better than reconstruction-guided suggestions and highlights one added value of the sweet spot. However, the difficulty of suggesting the best level or best contact with a sweet spot-guided approach is illustrated in Figure 5. In two out of three shown cases, the suggested best contacts appeared to be the best ones considering the location of the sweet spot, but the best clinical contacts were visually further away. More analysis may include side effects and sour spots to better understand these cases but were beyond the scope of the study.

Our suggestion performances for best level and best contact were lower than another sweet spot-guided study from our group (Jaradat et al., 2022). In that study, the suggested best level matched the best clinical level in 72% of the leads compared to 56% in the present study. We used the same sweet spot but with the visualization software Guide XT (Boston Scientific Corporation and Brainlab AG). In addition, more manual work to import the sweet spot and interaction with the software was needed to generate suggestions. For the suggestions in Guide XT, we aimed for a desired overlap volume of approximately 50% between VTA and sweet spot, since the software only allowed for a visual and qualitative inspection of the overlap. Moreover, the model to estimate VTAs in Guide XT is likely to be different from the instant analytical spheres model developed herein, though full details of the Guide XT model are not available.

In another comparison, our suggestion performances were similar to electrophysiology-guided suggestions. These were done with local field potentials recorded at our center and analyzed different frequency bands such as beta, gamma, or high-frequency oscillations (Tinkhauser et al., 2018; Shah et al., 2022). A possible explanation for the similar performance may be related to the location of the sweet spot, which is located at the dorsal border of the subthalamic nucleus. Two studies have shown that imaging and electrophysiology highly agree in detecting that dorsal border or entry into the subthalamic nucleus (Nowacki et al., 2018; Al Awadhi et al., 2022).

Comparisons to other studies using imaging-guided suggestions are not straightforward. Our suggestions here were compared to the best clinical levels and best clinical contacts from monopolar reviews. The suggestions themselves were not tested in patients for motor improvement and should therefore be viewed as an *indirect* validation. To our knowledge, four other studies tested imaging-guided suggestions in patients and found similar motor improvement compared to traditional clinical programming (Pourfar et al., 2015; Pavese et al., 2020; Lange et al., 2021; Waldthaler et al., 2021). These studies generated suggestions manually with a visualization software such as Guide XT but attempted to target the whole dorsolateral subthalamic nucleus and not a sweet spot. These studies can therefore be regarded as a *direct* validation, thus demonstrating the potential of imaging-guided suggestions. This warrants a future study with sweet spot-guided programming.

Imaging-guided suggestions can help reduce the time needed to program directional leads. For instance, Lange et al. (2021) reported an average of 20 min with imaging-guided programming compared to an average of 45 min with traditional programming for a patient implanted bilaterally with directional leads. Of note, they used the visualization in Guide XT to preselect a contact that faced the dorsolateral subthalamic nucleus. Then they determined the current amplitude for that contact with traditional programming, i.e., they started with 0 mA and slowly increased the current amplitude. Pavese et al. (2020) also targeted the dorsolateral subthalamic nucleus and additionally used VTA estimations, called stimulation field models in Guide XT, to suggest a current amplitude. Then they applied this amplitude and performed adjustments, when necessary. They reported an average of 36 min with imaging-guided programming compared to an average of 140 min with traditional programming for patients implanted bilaterally with octopolar leads. Our algorithm generated suggestions in a few seconds and could therefore produce similar time savings, which need to be verified. Shorter programming times could also be more comfortable for patients, who typically have to be off medication for programming.

Our suggestions for effect thresholds were acceptable *on average* with an error of 1.2 mA. But the suggested effect thresholds did not correlate well with the clinical effect thresholds. A possible explanation may be the sweet spot used herein. It was computed with clinical *efficiency*, i.e., input samples were normalized by the clinical effect threshold with the intention to highlight an effective and efficient region in the subthalamic nucleus (Nguyen et al., 2019). Therefore, samples with large clinical effect thresholds had less weight on the final sweet spot, whereas samples with small clinical effect thresholds had a larger weight on the final sweet spot. This might have flattened suggestions of effect thresholds. Overfitting may be another issue with voxel-wise sweet spots since these span thousands of voxels but have observations in the hundreds; though this is mitigated to an extent through voxel-wise statistics. These factors would need to be investigated in further analysis.

Nonetheless, suggested effect thresholds are likely to require clinical adjustments in each patient. For instance, Pavese et al. (2020) suggested current amplitudes that required adjustments in most of their patients. In contrast, Lange et al. (2021) did not use VTA estimations. In their view, these estimations made simplifying assumptions that would not adequately reflect the complexity of stimulated brain tissue. An *in-silico* study supports this point of view and reported differences between fast VTA estimations and more refined and computationally more expensive models (Duffley et al., 2019). Therefore, more work is needed to refine the suggestions for effect thresholds and to find a balance between added computation time and time for subsequent clinical adjustments (e.g., a complex computational model may take hours to calculate but need only minor adjustments, while a simpler model may take a few minutes but needs more adjustments).

Our study has several limitations. First, this analysis was performed with retrospective data. These were monopolar reviews done 4–6 months post-implantation with a focus on rigidity. A more comprehensive study would have included effects on bradykinesia or gait that were not measured in our data. We also did not consider side effects or sour spots in this study since our focus was on algorithm development. Second, the estimations of VTAs used herein have many limitations described in the literature (Duffley et al., 2019). Our estimations did not also consider stimulation frequency or pulse width. Third, an accumulation of errors from image acquisition, coregistration, normalization, and orientation detection might result in inaccuracies of the lead reconstructions.

### Outlook

DBS programming can be optimized and personalized to the symptom profile of the patient (Hollunder et al., 2022). This would leverage the full potential of directional DBS systems. One element toward personalization is symptom-specific sweet spots or fiber tracts, for instance for bradykinesia, tremor, or akinesia (Akram et al., 2017; Dembek et al., 2019; Hollunder et al., 2022). The sweet spot used here was specific to rigidity reduction. A second element is multipolar stimulation with current steering to target these different sweet spots or tracts while avoiding sour spots or tracts that are likely to cause side effects. The algorithm presented here is only capable of suggesting monopolar stimulation with one contact as the cathode and the implanted pulse generator as the return. A few *in-silico* studies have proposed algorithms to calculate multicathodic or multipolar suggestions and may be added to our algorithm in the future (Anderson et al., 2018; Pena et al., 2018; Cubo et al., 2019; Connolly et al., 2021; Roediger et al., 2022). Interestingly, Waldthaler et al. (2021) generated multipolar suggestions manually with the software Guide XT but had the dorsolateral part of the subthalamic nucleus as the only target.

## Conclusion

We have developed an algorithm to automatically suggest the best level, the best contact, and effect thresholds based on a sweet spot in the subthalamic nucleus. These sweet spot-guided suggestions can assist with the programming of directional DBS leads, in particular to suggest the best contacts. Moreover, an open-source graphical user interface was developed and is available with Lead-DBS. Further clinical analysis for a prospective study is warranted.

## Data availability statement

The datasets presented in this study can be found in online repositories. The names of the repository/repositories and accession number(s) can be found below: The source code for the graphical user interface and suggestion algorithms can be found at https://gitlab.switch.ch/brain-stimulation-mapping/automatic_dbs_stim_params_selection. The Lead-DBS reconstruction files of the 24 subjects are available at the University of Bern’s institutional repository BORIS Portal at https://doi.org/10.48620/120.

## Ethics statement

The studies involving human participants were reviewed and approved by the Kantonale Ethikkommission Bern (2020-02392). The patients/participants provided their written informed consent to participate in this study.

## Author contributions

SN: formal analysis, methodology, software, visualization, and writing—original draft. KP and AN: data curation and writing—review and editing. ID: data curation. PK: supervision and writing—review and editing. CP: conceptualization and supervision. TN: conceptualization, funding acquisition, supervision, and writing—review and editing. All authors contributed to the article and approved the submitted version.
